# Supplementary material for: West-Siberian Chernozem: How Vegetation and Tillage Shape Its Bacteriobiome
Source: Microorganisms. 2023 Sep 28;11(10):2431. doi: 10.3390/microorganisms11102431 (PMC10609427; doi:10.3390/microorganisms11102431)
Supplement: Supplementary file 1 [file microorganisms-11-02431-s001.zip › Naumova_et_ al_No-Till_Supplementary Table_S1.pdf]

microorganisms

MDPI

Supplementary material

Table S1. Relative abundance (%, mean) of the dominant bacterial orders in Chernozem of the experimental fields in the south of West Siberia

Taxon	Undisturbed		Ploughed		No till	
	0-5 cm	5-15 cm	0-5 cm	5-15 cm	0-5 cm	5-15 cm
Acidimicrobiales	2.6 [ab, 1]	2.4 [ab]	2.0 [a]	2.4 [ab]	2.0 [a]	3.0 [b]
Bacillales	1.3 [ab]	1.6 [b]	0.2 [a]	0.3 [a]	0.5 [a]	0.7 [ab]
Blastocatellales	1.0 [b]	0.2 [a]	1.2 [b]	0.4 [a]	0.9 [b]	0.3 [a]
Chitinophagales	1.0 [b]	0.2 [a]	0.5 [a]	0.3 [a]	1.1 [b]	0.4 [a]
Gaiellales	1.5 [a]	13.9 [c]	6.2 [b]	11.7 [c]	2.9 [a]	10.2 [c]
Gemmatimonadales	1.8 [b]	0.6 [a]	3.0 [c]	1.8 [b]	4.4 [c]	1.6 [b]
Geodermatophilales	0.1 [a]	0.0 [a]	3.8 [c]	1.5 [b]	5.6 [c]	1.6 [b]
Gp16	2.1 [a]	5.5 [b]	12.4 [c]	10.4 [c]	4.6 [ab]	4.3 [ab]
Gp3	0.4 [b]	0.1 [a]	1.0 [c]	0.6 [b]	1.0 [c]	0.4 [b]
Gp4	2.7 [c]	2.9 [c]	0.2 [a]	0.4 [a]	0.4 [a]	1.5 [b]
Gp6	10.9 [b]	7.1 [a]	12.4 [b]	14.8 [b]	7.3 [a]	13.6 [b]
Micromonosporales	6.5 [c]	1.0 [a]	1.1 [a]	0.7 [a]	2.5 [b]	1.1 [a]
Mycobacteriales	1.0 [b]	0.3 [a]	0.3 [a]	0.2 [a]	1.0 [b]	0.3 [a]
Myxococcales	2.1 [c]	0.4 [a]	1.6 [b]	0.9 [a]	2.3 [c]	0.7 [a]
Propionibacteriales	5.4 [a]	5.4 [a]	4.3 [a]	3.4 [a]	8.6 [b]	5.5 [a]
Pseudonocardiales	3.9 [c]	0.5 [a]	1.1 [a]	0.7 [a]	2.7 [b]	1.0 [a]
Rhizobiales	7.2 [b]	4.8 [ab]	3.2 [a]	2.9 [a]	3.9 [a]	3.6 [a]
Rhodospirillales	0.9 [b]	0.2 [a]	1.4 [c]	0.9 [b]	1.9 [c]	0.7 [b]
Rubrobacterales	3.7 [ac]	2.2 [a]	6.8 [c]	4.4 [ab]	5.7 [bc]	3.1 [ab]
Solirubrobacterales	10.1 [bc]	10.5 [c]	6.4 [a]	6.9 [a]	7.8 [ab]	8.1 [abc]
Spartobacteria_gis[2]	4.6 [b]	3.5 [b]	0.9 [a]	1.1 [a]	0.7 [a]	0.0 [a]
Sphaerobacterales	0.1 [a]	0.1 [a]	1.0 [b]	0.6 [b]	0.7 [b]	0.4 [b]
Sphingomonadales	0.1 [a]	0.0 [a]	0.7 [b]	0.1 [a]	1.0 [b]	0.0 [a]
un.[3] *Thermoleophilia*	2.6 [c]	4.6 [c]	1.0 [a]	1.8 [b]	0.8 [a]	2.3 [b]
WPS-1_gis	0.6 [ab]	0.3 [a]	0.8 [abc]	0.7 [abc]	1.1 [c]	0.5 [a]

[1] Different letters in rows indicate that the values are different ($P \leq 0.05$, Fisher's LSD test); [2] gis stands for genus incertae sedis; [3] un. stands for unclassified.
