# Supplementary material for: West-Siberian Chernozem: How Vegetation and Tillage Shape Its Bacteriobiome
Source: Microorganisms. 2023 Sep 28;11(10):2431. doi: 10.3390/microorganisms11102431 (PMC10609427; doi:10.3390/microorganisms11102431)
Supplement: Supplementary file 1 [file microorganisms-11-02431-s001.zip › Naumova_et_ al_No-Till_Supplementary Table_S2.pdf]

microorganisms

Supplementary material

Table S2. Relative abundance (%, mean) of the dominant bacterial families in Chernozem of the experimental fields in the south of West Siberia

Taxon	Undisturbed		Ploughed		No till	
	0-5 cm	5-15 cm	0-5 cm	5-15 cm	0-5 cm	5-15 cm
Bacillaceae1	1.0 [b, 1]	1.1 [b]	0.1 [a]	0.0 [a]	0.0 [a]	0.4 [ab]
Blastocatellaceae	0.4 [a]	0.1 [a]	1.0 [c]	0.4 [ab]	0.6 [b]	0.2 [a]
Bradyrhizobiaceae	2.0 [b]	0.2 [a]	0.3 [a]	0.5 [a]	0.4 [a]	0.5 [a]
Chitinophagaceae	1.0 [b]	0.2 [a]	0.5 [a]	0.3 [a]	1.1 [b]	0.4 [a]
Gaiellaceae	1.5 [a]	13.9 [c]	6.2 [b]	11.7 [c]	2.9 [a]	10.2 [bc]
Gemmatimonadaceae	1.8 [b]	0.6 [a]	3.0 [c]	1.8 [b]	4.4 [d]	1.6 [b]
Geodermatophilaceae	0.1 [a]	0.0 [a]	3.8 [c]	1.5 [b]	5.6 [c]	1.6 [b]
Iamiaceae	0.7 [a]	0.9 [a]	0.7 [ab]	1.0 [b]	0.6 [a]	1.1 [b]
Ilumatobacteraceae	0.9 [c]	0.2 [a]	0.9 [c]	0.4 [ab]	1.0 [c]	0.6 [b]
Kribbellaceae	1.0 [b]	1.0 [b]	0.4 [a]	0.5 [ab]	0.9 [b]	0.8 [ab]
Methylobacteriaceae	0.5 [b]	0.1 [a]	1.0 [c]	0.3 [ab]	1.9 [d]	0.4 [ab]
Micromonosporaceae	6.5 [c]	1.0 [a]	1.1 [a]	0.7 [a]	2.5 [b]	1.1 [a]
Mycobacteriaceae	1.0 [b]	0.3 [a]	0.2 [a]	0.1 [a]	0.6 [ab]	0.2 [a]
Nocardioidaceae	1.8 [b]	0.1 [a]	1.9 [bc]	0.4 [a]	2.5 [c]	0.4 [a]
Propionibacteriaceae	2.6 [a]	4.1 [ab]	1.9 [a]	2.4 [a]	5.1 [b]	4.1 [ab]
Pseudonocardiaceae	3.9 [c]	0.5 [a]	1.1 [a]	0.7 [a]	2.7 [b]	1.0 [a]
Rubrobacteraceae	3.7 [ab]	2.2 [a]	6.8 [b]	4.4 [ab]	5.7 [b]	3.1 [ab]
Solirubrobacteraceae	0.8 [a]	1.5 [a]	1.4 [a]	1.3 [a]	1.8 [a]	1.0 [a]
Sphaerobacteraceae	0.1 [a]	0.1 [a]	1.0 [c]	0.6 [b]	0.7 [bc]	0.4 [ab]
un.[2] *Acidimicrobiales*	0.9 [b]	1.4 [c]	0.4 [a]	1.0 [b]	0.4 [a]	1.4 [c]
un. *Myxococcales*	1.3 [bc]	0.2 [a]	1.0 [b]	0.5 [a]	1.4 [c]	0.3 [a]
un. *Rhizobiales*	4.3 [b]	4.2 [b]	1.5 [a]	1.8 [a]	0.9 [a]	2.3 [a]
un. *Solirubrobacterales*	9.1 [c]	8.9 [c]	4.8 [a]	5.5 [a]	5.8 [ab]	7.0 [bc]

[1] Different letters in rows indicate that the values are different (P≤0.05, Fisher's LSD test); [2] un. stands for unclassified.
